# Long-term safety and efficacy of ponesimod in participants with relapsing multiple sclerosis: results from the phase 3 OPTIMUM 5-year long term extension study

**DOI:** 10.1007/s00415-026-13675-7

**Published:** 2026-03-25

**Authors:** Xavier Montalban, Reinhard Hohlfeld, Carlo Pozzilli, Mark S. Freedman, Till Sprenger, Robert J. Fox, Eva Kubala Havrdova, Fred Lublin, Dan Huang, Nandini Raghavan, Janice Wong, Andrea Vaclavkova, Jelena Dukovski, Philippe Linscheid, Michel Burcklen, Ludwig Kappos

**Affiliations:** 1 Department of Neurology-Neuroimmunology, Multiple Sclerosis Center of Catalonia, Vall d’Hebron University Hospital, Barcelona, Spain; 2 Institute of Clinical Neuroimmunology, Ludwig Maximilians University Munich, Munich, Germany; 3 Munich Cluster of Systems Neurology (SyNergy), Ludwig Maximilians University Munich, Munich, Germany; 4 Sant’Andrea Multiple Sclerosis Centre, Sapienza University of Rome, Rome, Italy; 5 Department of Medicine, and The Ottawa Hospital Research Institute,, University of Ottawa, Ottawa, ON Canada; 6 Research Center for Clinical Neuroimmunology and Neuroscience Basel, Departments of Head Organs, Movement and Neuromedicine, Clinical Research, Biomedicine and Biomedical Engineering, University Hospital and University of Basel, Basel, Switzerland; 7 Universitätsspital Zürich, Zurich, Switzerland; 8 Cleveland Clinic, Cleveland, OH USA; 9 Department of Neurology, First Medical Faculty, Charles University, Prague, Czech Republic; 10 Icahn School of Medicine at Mount Sinai, New York, NY USA; 11 Johnson & Johnson Company, Neuss, Germany; 12 Johnson & Johnson, Titusville, NJ USA; 13 Actelion Pharmaceuticals, Part of Johnson & Johnson, Allschwil, Switzerland; 14 Janssen-Cilag GmbH, Neuss, Germany; 15 Research Center for Clinical Neuroimmunology and Neuroscience Basel, Departments of Head Organs, Movement and Neuromedicine, Clinical Research, Biomedicine and Biomedical Engineering, University Hospital and University of Basel, Spitalstrasse 2, CH-4031 Basel, Switzerland

**Keywords:** Long-term extension study, Ponesimod, Relapsing multiple sclerosis, Teriflunomide

## Abstract

**Introduction:**

In phase 3 OPTIMUM study, ponesimod demonstrated superior efficacy and acceptable safety vs teriflunomide in participants with relapsing multiple sclerosis (RMS). This long-term extension (LTE) study was designed to assess the safety and efficacy of ponesimod 20 mg (P20 mg) during extended treatment.

**Methods:**

Participants who completed core OPTIMUM study entered OPTIMUM-LTE; Participants receiving P20 mg once daily (OD) in core continued with the same dose (P20 mg/P20 mg) and those receiving teriflunomide 14 mg OD switched to P20 mg (T14 mg/P20 mg) in the LTE study. Safety assessments included treatment-emergent adverse events (TEAE). Efficacy was assessed using annualized relapse rate (ARR), time to first confirmed relapse up to end of study (EOS), confirmed disability accumulation (CDA), and magnetic resonance imaging (MRI)-based endpoints.

**Results:**

Of 1133 participants from core study, 877 were enrolled in 240-week LTE study (ponesimod: 439; teriflunomide:438). During LTE, 93.6% of participants in both groups experienced ≥ 1 TEAE; overall, serious TEAEs were experienced by 12.9% of participants (P20 mg/P20 mg: 12.8%; T14 mg/P20 mg: 13.0%) and TEAEs leading to study treatment discontinuation were experienced by 8.6% of participants (P20 mg/P20 mg: 7.7%; T14 mg/P20 mg: 9.4). In combined analysis period, mean ARR was 0.143 (95% CL: 0.123–0.167) for P20 mg/P20 mg and 0.184 (95% CL: 0.158–0.213) for T14 mg/P20 mg; 44.3% of participants in P20 mg/P20 mg and 49.5% in T14 mg/P20 mg experienced relapse. Overall, more participants in P20 mg/P20 mg vs T14 mg/P20 mg group achieved no evidence of disease activity (NEDA)-3 at end of LTE (17.5% vs 7.5%).

**Conclusions:**

Ponesimod demonstrated extended safety and sustained efficacy over 5 years in participants with RMS, without new safety signals. Results suggest that the effects on the MS disease control are maintained with ponesimod over the LTE treatment period.

**Supplementary Information:**

The online version contains supplementary material available at 10.1007/s00415-026-13675-7.

## Introduction

Multiple sclerosis (MS) is a chronic, progressive neurological disease mediated by immune responses that cause demyelination, neurodegeneration, and recurrent inflammation in the central nervous system, often resulting in significant disability. Over time, MS can lead to irreversible clinical and cognitive impairments [1]. The long-term impact of MS includes ongoing disability progression and neurodegeneration, which can significantly affect participants' quality of life. Therefore, long-term treatments are essential to manage disease activity, reduce relapses, and slow disability progression, ultimately improving long-term outcomes and preserving function over time [2].

Ponesimod is a second generation, orally active, selective modulator of the sphingosine 1-phosphate receptor 1 (S1P1) that exhibits rapid, dose-dependent, and reversible effects [3]. By selectively targeting S1P1, ponesimod reduces the migration of immune cells primarily T and B lymphocytes—across the blood–brain barrier, thereby reducing central nervous system inflammation associated with autoimmune conditions [4, 5]. Unlike other S1P modulators, ponesimod is highly selective for S1P1 and is rapidly eliminated allowing for more targeted, rapid, and reversible immunomodulation along with a maximum reduction in lymphocyte count [6, 7]. Ponesimod offers certain advantages over other S1P receptor modulators due to its pharmacokinetic and pharmacodynamic profile. Ponesimod’s short half-life (~ 33 h) allows for rapid drug elimination and swift recovery of peripheral lymphocyte counts upon discontinuation. This may facilitate management of adverse events, reduce the risk of prolonged immunosuppression-related adverse events (e.g., serious infections), and provide flexibility in treatment planning, including pregnancy considerations.

The OPTIMUM study was the first phase 3 superiority study comparing two oral disease-modifying therapies for relapsing multiple sclerosis (RMS). The efficacy of ponesimod was evaluated against the dihydroorotate dehydrogenase (DHODH) inhibitor teriflunomide [8]. The OPTIMUM study showed that ponesimod significantly (*p* < 0.001) reduced the annualized relapse rate (ARR) by 30.5% compared with teriflunomide; it also decreased active MRI lesions and brain volume loss, both established measures of MS pathology. At week 108, ponesimod significantly reduced and lowered the annual rate of active MRI lesions by 56% compared to teriflunomide. No significant difference was observed in time to 12- or 24-week confirmed disability accumulation (CDA). Ponesimod has demonstrated an acceptable safety profile in phase 3, OPTIMUM core study and phase 2 core and extension study [8, 9]. Based on the findings of a placebo-controlled phase 2b study [10] and the results of the OPTIMUM study [8], ponesimod was approved by the U.S. Food and Drug Administration (FDA) in March 2021, European Commission (EC) across 27 member states in May 2021, and in Australia in March 2022 for treatment in adults with relapsing forms of MS [11–13].

This long-term extension (LTE) study of OPTIMUM was designed to further assess the safety and efficacy of ponesimod 20 mg (P20) during an extended treatment period in participants with RMS. Additionally, the study examined the impact on disease activity (relapse and MRI-detected MS lesions) of an approximate 14-day treatment interruption between the core and reinitiation of treatment in the extension phases. Finally, the safety and efficacy of switching from teriflunomide 14 mg (T14) to P20 were also evaluated. Given that patients in the T14 mg/P20 mg group switched treatment at the start of the extension phase and that baseline characteristics may not be comparable between groups at LTE entry, all efficacy analyses were conducted descriptively, without formal statistical comparisons.

## Methods

### Study design

This was a prospective, multicenter, open-label, non-comparative, LTE of the phase 3 OPTIMUM study (NCT02425644) [8]. In the double-blind core study, participants were randomized (1:1) to receive ponesimod 20 mg or teriflunomide 14 mg once daily [8]. Participants who completed the 108-week double-blind treatment period had the option to enter the LTE study after completing the safety follow-up period of the core study. Eligible participants receiving ponesimod 20 mg once daily in the core study continued to receive ponesimod 20 mg in the LTE (P20 mg/P20 mg group) and those receiving teriflunomide 14 mg switched to receive ponesimod 20 mg in the LTE (T14 mg/P20 mg group). An accelerated treatment elimination procedure was required by the protocol in alignment with the approved teriflunomide label at the end of the core study for all participants entering the LTE [12]. For participants who transitioned into the LTE, the core study safety follow-up period concluded with a safety follow-up visit 1 or with an abbreviated follow-up visit 2 at 14 to 22 or 23 to 37 days, respectively, after the last dose of the study drug (end-of-treatment [EOT]) in the core study (Supplementary Fig. 1).

This LTE study was divided into three distinct periods: a pretreatment period which included all pre-dose assessments from signing of the informed consent until the first dosing of ponesimod treatment of the extension study; a treatment period (up to 240 weeks); and a safety follow-up (Supplementary Fig. 1). The treatment period consisted of an up-titration period from day 1 to day 14 (ponesimod dose escalation from 2 to 10 mg per day) and a maintenance period from day 15 until end of treatment (ponesimod 20 mg per day). The treatment period lasted for 240 weeks or until ponesimod was commercially available for the treatment of MS in the participant’s country except Ukraine, where the treatment period was permitted to last for up to 288 weeks (due to a regional crisis). Study treatment duration in some countries may have been shortened (< 240 weeks) due to ponesimod becoming commercially available.

### Participants

Participants who completed the 108-week treatment period in the phase 3 OPTIMUM core study were eligible to enter the LTE study. Baseline characteristics such as age (18–55 years), diagnosis of relapsing MS (including RRMS or SPMS with relapses per 2010 McDonald criteria), Expanded Disability Status Scale (EDSS) score of 0–5.5, and evidence of recent clinical or MRI activity applied to enrollment in the core study, not the LTE. Sufficient compliance with the accelerated teriflunomide elimination procedure was assessed by the investigator at follow-up visit 1 or abbreviated follow-up visit 2 of the core study, whichever occurred last. All details of eligibility criteria for the core study were previously summarized [8].

### Study evaluations

#### Safety

Safety assessments included the incidence of treatment emergent adverse events (TEAE), serious TEAEs, TEAEs of special interest (associated with known ponesimod AEs) and adverse events (AEs) leading to premature discontinuation of treatment. Safety was also assessed using clinical laboratory tests, which included hematology, blood chemistry and urinalysis, as well as 12-lead electrocardiography (ECG), blood pressure, weight, spirometry, DLCO tests (substudy), ophthalmological and dermatological examination.

#### Efficacy

##### Clinical endpoints

All efficacy endpoints of this LTE were exploratory in nature and these included ARR, defined as the number of confirmed relapses per patient-year. A relapse was defined as new, worsening, or recurrent neurological symptoms that occurred ≥ 30 days after the previous relapse and lasted for at least 24 h in the absence of fever or infection. Additionally, time from core baseline to first 12- or 24-week CDA (defined as an increase in the EDSS, which was confirmed after 12/24 weeks, by at least 1.5 with a baseline EDSS score of 0.0, at least 1.0 with baseline EDSS score of 1.0 to 5.0, or at least 0.5 with a baseline EDSS score of 5.5 or more), change from baseline to end of study (EOS was reached when end of treatment and subsequent safety follow-up were completed) in EDSS scores, and no evidence of disease activity (NEDA) status at end of study according to NEDA 3 (composite of no relapse, no 12-week CDA, no Gd + T1 or new or enlarging T2 lesions) and NEDA 4 criteria (a composite of NEDA-3 and no brain volume annual rate decrease of ≥ 0.4% from baseline to EOS) [14, 15].

##### Magnetic resonance imaging (MRI)-based endpoints

These included combined unique active lesions (CUAL) and cumulative number of CUALs (CUAL was defined as all new gadolinium-enhancing (Gd +) T1 lesions and all new or enlarging T2 lesions, without double counting of lesions), total number of Gd + T1 lesions, cumulative number of new or enlarging T2 lesions, volume of T1 and T2 lesions, absence of T1 and T2 lesions, proportion of Gd + T1 lesions at baseline evolving to persistent black holes (PBHs), and percent change from baseline in brain volume.

##### Other endpoints

The other endpoints were change from baseline in Multiple Sclerosis Functional Composite (MSFC) Z-score assessing Timed 25-Foot Walk (lower extremity function), 9-Hole Peg Test ( upper extremity function), and Paced Auditory Serial Addition Test (PASAT), and change in the Symbol Digit Modalities Test (SDMT, [both measuring attention and processing speed]).

### Statistical analysis

#### Sample size

The sample size of this study was determined by the number of participants who completed the OPTIMUM core study and subsequently consented to enter the LTE study [8].

### Analysis periods

#### Combined analysis period

This period included all available data from randomization in the core study up to the extension EOS for participants entering the extension. For safety, this period included all available data from the date of first treatment administration in the core study up to the last treatment date in the extension study + 15 days for participants entering the extension, or until the last treatment date in the core study + 15 days for participants not entering the extension.

#### Extension analysis period

This period included all available data collected on or after the first intake of ponesimod treatment in the extension study through the extension EOS date for efficacy variables, or the last treatment date in the extension study + 15 days for safety variables. This definition applied regardless of whether the participants received commercially available ponesimod after completing study treatment.

### Analysis sets

#### Extension set

The extension analysis set (EXTS) included all participants who provided written informed consent to enter the extension study and who received at least 1 dose of ponesimod study treatment in the extension study.

The safety and efficacy endpoints of this study were exploratory and were analyzed for the extension analysis period (LTE only) and combined analysis period (core OPTIMUM plus LTE studies) respectively, with no multiplicity adjustments being made for the efficacy endpoints. Safety was evaluated in participants (439 in P20 mg/P20 mg and 438 in the T14 mg/P20 mg) who received at least 1 dose of ponesimod in the extension study and were included in the EXTS set. For safety analyses, participant data were grouped and summarized by core study treatment groups and the whole safety analysis group, using the extension baseline as the reference. All safety analyses presented here are for the extension analysis period, extension set. Efficacy analyses were used to interpret the disease activity observed in participants after long-term treatment with ponesimod 20 mg and to understand the impact of switching to ponesimod 20 mg from teriflunomide 14 mg using the core OPTIMUM study baseline as a reference. A negative binomial (NB) model for estimating the ARR of confirmed relapses was used, with core study treatment as a factor and the logarithm of time on study (in years) as an offset variable. Mean model-based estimates of the ARR for confirmed relapses, by core study treatment group, as well as 95% confidence intervals (CIs) are presented. A rate ratio, including 95% CIs, comparing ponesimod with teriflunomide 14 mg was derived from the model.

All efficacy and safety analyses in the LTE were descriptive. No formal statistical comparisons were conducted between the P20 mg/P20 mg and T14 mg/P20 mg groups, due to the non-randomized design, treatment switching in the extension analysis period, and differences in baseline characteristics at LTE entry. As a result, only descriptive statistics are reported.

## Results

### Participant disposition, baseline characteristics and time in extension study

The core OPTIMUM study enrolled a representative relapsing MS population (median age 37 years; range 18–55), with 64.9% female participants. Among the 1131 patients who received treatment, 83.1% in the ponesimod group and 83.6% in the teriflunomide group completed the 108-week core study. Detailed demographic and baseline clinical characteristics of the study population have been published previously in the primary OPTIMUM study.[8] Of the 1,133 participants enrolled in the core OPTIMUM study, 877 (439 [77.4%] on ponesimod 20 mg and 438 [77.4%] on teriflunomide 14 mg) were enrolled in the 240-week LTE study, and all participants were treated with ponesimod 20 mg once daily (Supplementary Fig. 2). The median (range) time in the extension study was 56.21 (0.7, 70.9) months for P20 mg/P20 mg and 56.20 (1.0, 73.2) months for T14 mg/P20 mg. A total of 223 participants (25.4%) prematurely discontinued study treatment during the LTE study (106 [24.1%] in P20 mg/P20 mg and 117 [26.7%] in T14 mg/P20 mg; Supplementary Fig. 1). The primary reasons for premature discontinuation of study treatment during the LTE study were withdrawal of consent by participants (53 [12.1%] in P20 mg/P20 mg and 72 [16.4%] in T14 mg/P20 mg), which included AEs (6 [1.4%] in P20 mg/P20 mg and 14 [3.2%] in T14 mg/P20 mg) and lack of efficacy (10 [2.3%] in P20 mg/P20 mg and 19 [4.3%] in T14 mg/P20 mg; Table 1**)**.

**Table 1:** Reasons for premature discontinuation from extension study

	P20 mg/P20 mg *n* = 439	T14 mg/P20 mg *n* = 438
Participants who prematurely withdrew from the extension study	**87 (19.8)**	**67 (15.3)**
Reason for premature withdrawal from the extension study		
Death	1 (0.2)	0
Lost to follow-up	6 (1.4)	6 (1.4)
Withdrawal of consent	**67 (15.3)**	**54 (12.3)**
Adverse event, n (%)	4 (0.9)	7 (1.6)
Lack of efficacy, n (%)	9 (2.1)	8 (1.8)
No reason provided	16 (3.6)	15 (3.4)
Other	38 (8.7)	24 (5.5)
Physician decision	13 (3.0)	7 (1.6)
Adverse event, n (%)	3 (0.7)	5 (1.1)
Lack of efficacy, n (%)	1 (0.2)	0
Other	9 (2.1)	2 (0.5)

All values are expressed as n (%)

All participants (including those randomized to Teriflunomide treatment group at core study) received ponesimod 20 mg in Extension analysis period

Bold values indicate the total number affected

 Overall, the majority of participants were white (97.9%), with women accounting for around two thirds (65.7%) of participants and a median age of 39.0 years at the start of the extension study (range: 20–58 years; Table 2).

**Table 2:** Baseline characteristics

	P20 mg/P20 mg *n* = 439	T14 mg/P20 mg *n* = 438	Total *N* = 877
Sex, Female, n (%)	286 (65.1)	290 (66.2)	576 (65.7)
Age, Mean (SD), years	38.8 (8.77)	39.4 (8.78)	39.1 (8.77)
Race, n (%)			
Black or African American	2 (0.5)	2 (0.5)	4 (0.5)
White	429 (97.7)	430 (98.2)	859 (97.9)
Other	4 (0.9)	2 (0.5)	6 (0.7)
Not applicable	4 (0.9)	4 (0.9)	8 (0.9)
BMI (kg/m^2^)			
Mean (SD)	24.7 (5.1)^c^	24.4 (4.8)^c^	24.6 (5.0)^e^
Median (Min, Max)	23.6 (15.6, 45.1)^c^	23.6 (14.8, 46.3)^c^	23.6 (14.8, 46.3)^e^
BMI, by category, n (%)			
n	437	437	874
< 18.5 kg/m^2^	25 (5.7)	26 (5.9)	51 (5.8)
≥ 18.5 – < 25 kg/m^2^	236 (54.0)	248 (56.8)	484 (55.4)
≥ 25 – < 30 kg/m^2^	105 (24.0)	112 (25.6)	217 (24.8)
≥ 30 kg/m^2^	71 (16.2)	51 (11.7)	122 (14.0)

*BMI* Body mass index, SD Standard deviation

*P20 mg/P20 m* Ponesimod 20 mg/Ponesimod 20 mg, *T14 mg/P20 mg* Teriflunomide 14 mg/Ponesimod 20 mg

^a^n = 438

^b^n = 876

^c^n = 437

^d^n = 875

^e^n = 874

### Safety outcomes

During the LTE study, 93.6% of participants in both treatment groups each experienced at least one TEAE (Table 3). Serious TEAEs were experienced by 12.9% of participants overall (12.8% in P20 mg/P20 and 13.0% in T14 mg/P20). SAE were mostly isolated with no observed pattern or clustering in either group. The most common system organ class (SOCs) were Infections and Infestations (3.3%), Surgical/Medical procedures (1.6%), and Nervous system disorders (1.6%). One death was reported in the P20 mg/P20 mg dose group due to pulmonary embolism, venous thrombosis and was considered not related to the study drug by the investigator. A slightly lower proportion of participants in the P20 mg/P20 mg dose group reported drug discontinuation due to TEAEs than in the T14 mg/P20 mg dose group, 7.7% vs 9.4%, respectively. The most commonly reported AEs leading to premature discontinuation of study treatment were unintended pregnancy (1.5%), alanine aminotransferase (ALT) increased (0.8%), dyspnea (0.6%), and macular edema (0.6%), all of which being protocol-mandated study-specific criteria for premature discontinuation of study treatment. An AE of lymphopenia leading to discontinuation of study treatment was reported for 3 participants (2 in P20 mg/P20 and 1 in T14 mg/P20 group) without associated infections. Protocol mandated criteria for discontinuation included the confirmed total lymphocyte threshold count of < 0.2 × 109/L (< 200 cells/mm3). AEs within the Infection and Infestation SOC, that lead to study treatment discontinuation were 3 in P20 mg/P20 group (Hepatitis B, Hepatitis E and Oral herpes, 1 each) and 1 Lyme disease in the T14 mg/P20 group. The most commonly reported TEAEs (≥ 15% of participants in any group) by preferred terms (PT) in the LTE study were COVID-19 and increased ALT levels, (Table 4). Regarding the liver enzyme elevation, study protocol followed the FDA guidance (2009) for close monitoring for drug-induced liver injury (DILI). After thorough review of all increased LFTs and related reported PTs, no DILI case was identified.

**Table 3:** Overview of TEAEs during the extension study period

	P20 mg/P20 mg *n* = 439	T14 mg/P20 mg *n* = 438	Total *N* = 877*
Participants with at least one TEAE n (%)	411 (93.6)	410 (93.6)	821 (93.6)
TEAE leading to study drug discontinuation n (%)	34 (7.7)	41 (9.4)	75 (8.6)
Serious TEAE n (%)	56 (12.8)	57 (13.0)	113 (12.9)
Fatal TEAE n (%)	1 (0.2)	0 (0)	1 (0.1)

*TEAE* Treatment-emergent adverse event

*P20 mg/P20 mg* Ponesimod 20 mg/Ponesimod 20 mg, *T14 mg/P20 mg* Teriflunomide 14 mg/Ponesimod 20 mg

^*^All participants received ponesimod 20 mg in extension study period

**Table 4:** TEAEs reported in ≥ 5% of study participants during the extension study period

	P20 mg/P20 mg *n* = 439	T14 mg/P20 mg *n* = 438	Total *N* = 877
Participants with ≥ 1 TEAEs	**411 (93.6)**	**410 (93.6)**	**821 (93.6)**
COVID-19	116 (26.4)	108 (24.7)	224 (25.5)
Nasopharyngitis	82 (18.7)	74 (16.9)	156 (17.8)
ALT increased	73 (16.6)	98 (22.4)	171 (19.5)
Lymphopenia	66 (15.0)	64 (14.6)	130 (14.8)
Headache	57 (13.0)	64 (14.6)	121 (13.8)
Upper respiratory tract infection	47 (10.7)	51 (11.6)	98 (11.2)
Back pain	45 (10.3)	30 (6.8)	75 (8.6)
Hypertension	37 (8.4)	44 (10.0)	81 (9.2)
Urinary tract infection	36 (8.2)	33 (7.5)	69 (7.9)
Arthralgia	31 (7.1)	38 (8.7)	69 (7.9)
Lymphocyte count decreased	30 (6.8)	30 (6.8)	60 (6.8)
Fatigue	24 (5.5)	30 (6.8)	54 (6.2)
Respiratory tract infection	23 (5.2)	15 (3.4)	38 (4.3)
Diarrhea	22 (5.0)	15 (3.4)	37 (4.2)
Dizziness	22 (5.0)	17 (3.9)	39 (4.4)
Leukopenia	18 (4.1)	23 (5.3)	41 (4.7)
AST increased	17 (3.9)	26 (5.9)	43 (4.9)

*AE* Adverse event, *ALT* Alanine aminotransferase, *AST* Aspartate aminotransferase, *TEAE* Treatment-emergent adverse events

*P20 mg/P20 mg* Ponesimod 20 mg/Ponesimod 20 mg, *T14 mg/P20 mg* Teriflunomide 14 mg/Ponesimod 20 mg

^*^All participants received ponesimod 20 mg in extension study period

Bold values indicate the total number affected

The most commonly reported TEAEs by SOC were Infections and infestations (66.3% in P20 mg/P20 mg and 62.3% in T14 mg/P20 mg), Investigations (36% vs 40%, respectively) and Nervous system disorders (28.9% vs 31.1%, respectively). The most commonly reported PTs (≥ 5% of participants in any group) within the Nervous system disorder SOC were headache (13% in P20 mg/P20 mg and 14.6% in T14 mg/P20 mg) and dizziness (5% vs 3.9%). No posterior reversible encephalopathy syndrome (PRES) and no acute disseminated encephalomyelitis **(**ADEM) were reported. With regards to the Infections, no progressive multifocal leukoencephalopathy (PML) case and no serious opportunistic infections were reported in the study.

### Efficacy outcomes

#### Annualized relapse rate (ARR)

**Fig. 1:**
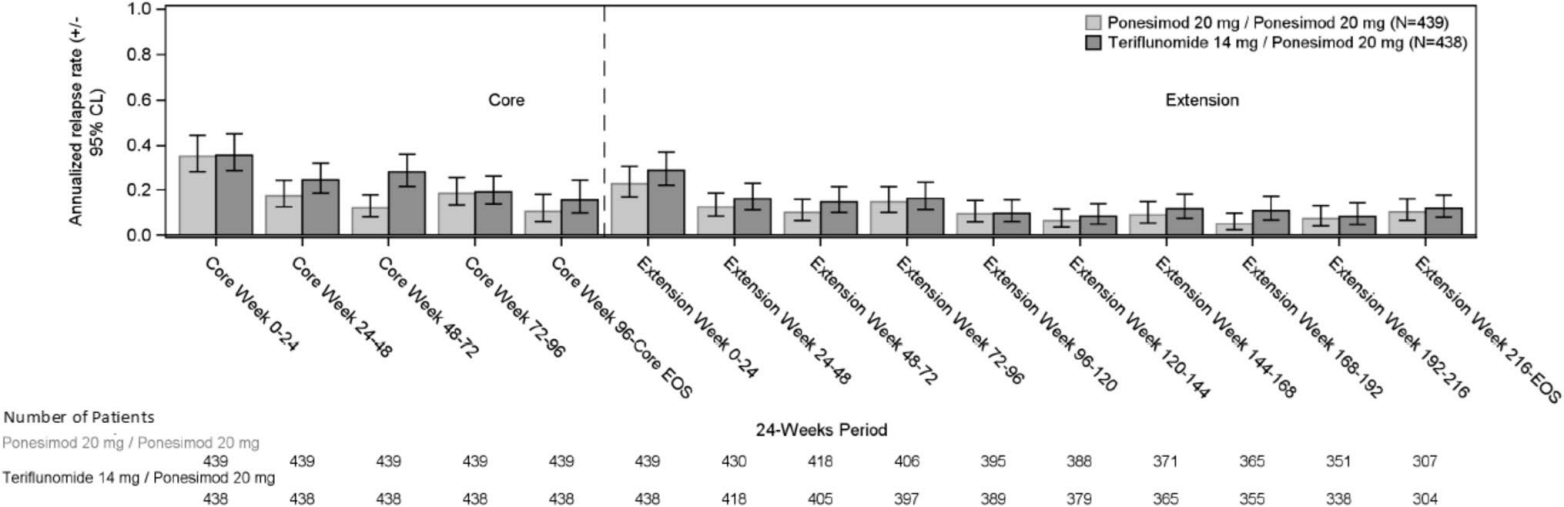
Annualized relapse rates over time in the P20 mg/P20 mg and T14 mg/P20 mg groups. EOS is defined as end of extension study or cut-off date. 24-week period is calculated relative to randomization in the core study or core EOS/extension study start as applicable. The last interval 'Week 216—EOS' can be longer than 24 weeks. It contains relapse from extension week 216 to EOS for all participants (including the Ukrainian participants where extended treatment periods were allowed). All participants (including participants randomized to Teriflunomide treatment group at core study) received ponesimod 20 mg in Extension analysis period. ARR, annualized relapse rate

The mean ARR for confirmed relapses over the combined analysis period estimated from negative binomial regression model was 0.143 (95% CL: 0.123–0.167) for the P20 mg/P20 mg group and 0.184 (95% CL: 0.158–0.213) for the T14 mg/P20 mg group. The treatment effect over the combined analysis period (rate ratio P20 mg/P20 mg versus T14 mg/P20 mg) up to the EOS was 0.779 (95% CL: 0.629–0.965). The mean (SD) number of confirmed relapses up to EOS per participant was 0.856 (1.451) in the P20 mg/P20 mg group and 1.091 (1.621) in the T14 mg/P20 mg group, with most participants having < 2 confirmed relapses (Fig. 1).

#### Time to first confirmed relapse, duration of relapse and relapse characteristics

**Fig. 2:**
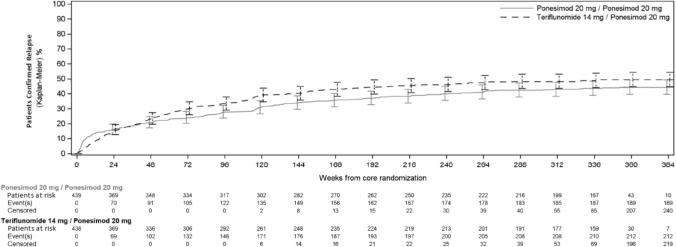
Time to first confirmed relapse (weeks). All participants (including participants randomized to the Teriflunomide treatment group at the core study) received ponesimod 20 mg in the Extension analysis period

Kaplan–Meier estimate for the percentage of participants who had experienced a confirmed relapse by week 384 in the combined analysis period was 44.3% (95% CL: 39.7–49.3) in the P20 mg/P20 mg group and 49.5% (95% CL: 44.8–54.4) in the T14 mg/P20 mg group **(**Fig. 2**).** Over the combined analysis period on the EXTS set, the total number of confirmed relapses up to EOS was 376 in the P20 mg/P20 mg group and 478 in the T14 mg/P20 mg group, with 95.5% and 96.7%, respectively, requiring treatment with corticosteroids. The median duration of per protocol confirmed relapses over the course was 21.0 days in the P20 mg/P20 mg group (*n* = 360 relapses with available data of duration) and 22.0 days in the T14 mg/P20 mg group (*n* = 463 relapses with available data of duration). Additionally, 56.7% of participants in the P20 mg/P20 mg group and 51.6% of participants in the T14 mg/P20 mg groups remained relapse-free from core study baseline to end of the LTE study.

#### Confirmed disability accumulation (CDA)

Over the combined analysis period, the Kaplan–Meier estimated proportion of participants experiencing 12-week CDA up to week 396 was 23.1% (95% CL: 19.3–27.5) in the P20 mg/P20 mg group and 30.5% (95% CL: 26.2–35.3) in the T14 mg/P20 mg group. Similar results were observed for 24-week CDA between the two groups by Week 384, with 21.3% (95% CL: 17.7–25.6) of participants in the P20 mg/P20 mg group and 26.0% (95% CL: 21.9–30.6) of participants in the T14 mg/P20 mg group.

#### Change from baseline in EDSS, NEDA-3, and NEDA-4 status

**Fig. 3:**
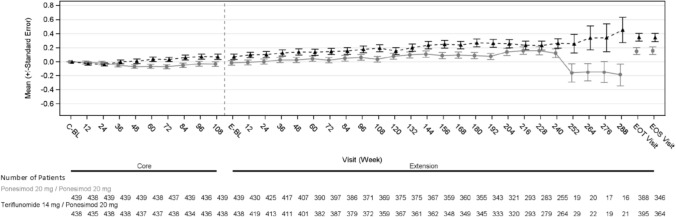
Change in EDSS score over time. All participants (including participants randomized to Teriflunomide treatment group at core study) received ponesimod 20 mg in Extension analysis period. Intervals up to the time point where there were at least 5 participants of each arm. The Extension Week 240 contains data of this visit of Ukrainian participants with allowed extended treatment time and data of EOT visit planned at week 240 of other participants. The Extension EOT visit contains data of all EOT visits of treatment completers and participants with early treatment discontinuation irrespective of time of discontinuation. The post-week 240 visits include Ukrainian participants only. *EDSS* Expanded disability status scale, *SE* standard error

The mean absolute EDSS scores over the combined analysis period were observed to increase over time, with numerically slightly higher values observed in the T14 mg/P20 mg group compared with the P20 mg/P20 mg group (Fig. 3). At extension baseline, mean EDSS remained similar to core Week 108 in both treatment groups. At the EOS Visit, the mean EDSS score was 2.56 (median: 2.00) in the P20 mg/P20 mg group (*n* = 346) and 2.80 (median: 2.50) in the T14 mg/P20 mg group (*n* = 364), and mean (SD) absolute change from core baseline was 0.16 (1.008) in the P20 mg/P20 mg group and 0.34 (1.105) in the T14 mg/P20 mg group.

Over the combined analysis period from core Baseline to EOS, 17.5% of participants in the P20 mg/P20 mg dose group and 7.5% of participants in the T14 mg/P20 mg dose group achieved NEDA-3 status **(**Table 5**).** The most frequent reason for not achieving NEDA-3 status at extension EOS was the presence of new or enlarging T2 lesions (65.1% in P20 mg/P20 mg and 76.3% in T14 mg/P20 mg). Over the combined analysis period, 5.2% of participants in the P20 mg/P20 mg dose group and 2.3% of participants in the T14 mg/P20 mg dose group achieved NEDA-4 status **(**Table 5**).** The most common reason for not achieving NEDA-4 in these participants was an annual rate of brain volume decrease ≥ 0.4% from baseline (73.6% in P20 mg/P20 mg and 82.6% in T14 mg/P20 mg) and the presence of new or enlarging T2 lesions (65.1% in the P20 mg/P20 mg group and 76.3% in the T14 mg/P20 mg group).

**Table 5:** NEDA-3 and NEDA-4 status (combined analysis period)

	P20 mg/P20 mg *n* = 439	T14 mg/P20 mg *n* = 438
NEDA-3 status at EOS		
Yes, n (%)	77 (17.5)	33 (7.5)
No, n (%)	362 (82.5)	403 (92.0)
NEDA-4 status at EOS		
Yes, n (%)	23 (5.2)	10 (2.3)
No, n (%)	414 (94.3)	425 (97.0)

*EOS* End of study, *NEDA* No evidence of disease activity

All values are expressed as n (%). All participants (including those randomized to the Teriflunomide treatment group at the core study) received ponesimod 20 mg in the Extension analysis period

### MRI-based endpoints

#### Absence of Gd + T1 lesions

At the core baseline, more than half of participants had no Gd + T1 lesions (P20 mg/P20 mg group = 59.5% T14 mg/P20 mg group = 55.7%). From core baseline up to extension EOT, at each scheduled and unscheduled MRI visit from core baseline up to end of LTE study, 66.7% of participants in the P20 mg/P20 mg group and 54.3% in the T14 mg/P20 mg group had no new Gd + T1 lesions.

#### Total number of new or enlarging T2 lesions

The mean number of new or enlarging T2 lesions per visit decreased in both treatment groups during the extension study. The mean number of new or enlarging T2 lesions per visit decreased from extension Week 48 (1.704 [95% CL: 1.373–2.116] in P20 mg/P20 mg [*n* = 406] and 1.758 [95% CL: 1.412–2.188] in T14 mg/P20 mg [*n* = 396]) to extension week 96 (0.997 [95% CL: 0.767–1.296] in P20 mg/P20 mg [*n* = 367] and 0.959 [95% CL: 0.736–1.248] in T14 mg/P20 mg [*n* = 364]).

#### Cumulative number of new or enlarging T2 lesions

Using an NB regression model with core study treatment as a factor, the cumulative number of new or enlarging T2 lesions over the combined analysis period on the EXTS was 1.352 (95% CL: 1.152–1.586) in the P20 mg/P20 mg group and 1.951 (95% CL: 1.664–2.287) in the T14 mg/P20 mg group.

#### Combined unique active lesions (CUALs)

**Fig. 4:**
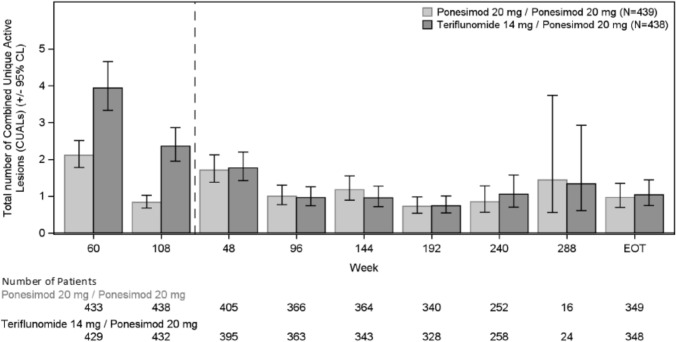
Changes in CUALs over time. All participants (including participants randomized to Teriflunomide treatment group at core study) received ponesimod 20 mg in the Extension analysis period. A negative binomial model without offset is applied with Wald confidence intervals. The Extension Week 240 contains data of this visit of Ukrainian participants with allowed extended treatment time and data of the EOT visit planned at week 240 of other participants. The Extension EOT visit contains data of all EOT visits of treatment completers and participants with early treatment discontinuation. The post-week 240 visits are applicable for Ukrainian participants only. *CUALS* Combined unique active lesions

The number of CUALs over the combined analysis period on the EXTS was observed to decrease over time (Fig. 4). The number of participants with 0 CUALs at extension EOT Visit was 265 (75.9%) in the P20 mg/P20 mg group (*n* = 349) and 272 (78.2%) in the T14 mg/P20 mg group (*n* = 348), compared with 254 (62.7%) in the P20 mg/P20 mg group (*n* = 405) and 229 (58.0%) in the T14 mg/P20 mg group (*n* = 395) at extension Week 48. Using an NB regression model with core study treatment as a factor, the mean cumulative CUALs per year were 1.352 (95% CL: 1.153–1.586) in the P20 mg/P20 mg group and 1.954 (95% CL: 1.667–2.291) in the T14 mg/P20 mg group. The proportion of participants who did not have any CUALs through the combined analysis period was 34.7% in the P20 mg/P20 mg group and 23.2% in the T14 mg/P20 mg group.

#### Brain volume

**Fig. 5:**
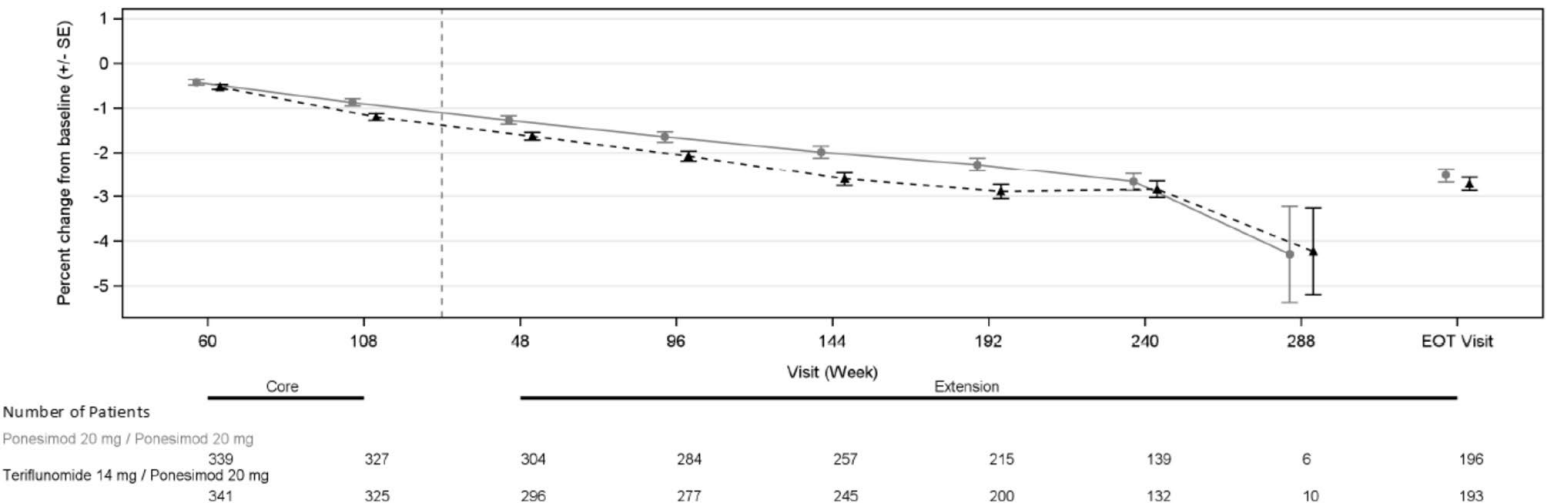
Changes in brain volume over time. All participants (including participants randomized to the Teriflunomide treatment group at the core study) received ponesimod 20 mg in the Extension analysis period. Intervals up to the time point where there are at least 5 participants in each arm. The Extension Week 240 contains data of this visit of Ukrainian participants with allowed extended treatment time and data of the EOT visit planned at week 240 of other participants. The Extension EOT visit contains data of all EOT visits of treatment completers and participants with early treatment discontinuation. The post-week 240 visits are applicable for Ukrainian participants only

A gradual, comparable decrease in mean brain volume from core baseline to LTE study EOT was observed over time in both treatment groups (Fig. 5). At the extension EOT Visit, the mean (SD) percent change from core baseline in the annual rate of brain volume was –2.52% (SD 2.179) in the P20 mg/P20 mg dose group compared with − 2.72% (SD 2.024) in the T14 mg/P20 mg dose group.

### Other endpoints

#### MSFC Z-scores and SDMT scores

The mean changes over the combined analysis period on the EXTS in MSFC Z-scores were similar between the two treatment groups at the end of the LTE study period. At the extension EOT, the mean (SD) absolute change from core baseline MSFC Z-score was –0.001 (0.6457) in the P20 mg/P20 mg dose group and –0.210 (0.8443) in the T14 mg/P20 mg dose group. Similarly, over the combined analysis period, the mean change from baseline in SDMT scores was similar between the two treatment groups. At the extension EOT, the mean (SD) absolute change from core baseline SDMT score was 0.0 (16.30) in the P20 mg/P20 mg dose group and –0.6 (15.97) in the T14 mg/P20 mg dose group.

## Discussion

LTE studies in MS provide valuable data on the continued effectiveness of treatments, help identify late-emerging adverse effects, and may guide clinical decision-making for lifelong management of MS [9, 16]. The safety profile of ponesimod was consistent across both groups, with the incidence of TEAEs being comparable in the continuous ponesimod and switch groups. The proportion of participants in both treatment groups who experienced at least one TEAE was comparable. The overall rates of discontinuation due to TEAEs were low in both groups. The most common TEAEs, including COVID-19, nasopharyngitis, and increased ALT levels, were consistent with the safety profiles of other S1P modulators and MS therapies as well as those observed in the core OPTIMUM study and phase 2b core and extension studies [9, 10]. This finding aligns with the known safety profile of ponesimod and other S1P receptor modulators, suggesting that switching to ponesimod does not introduce new safety risks. The incidence of infections and Nervous System Disorders was also consistent with previous data from studies of ponesimod. In terms of efficacy, results from the 5-year LTE of the OPTIMUM study support the sustained effect of ponesimod on disease activity in participants with RMS who received up to 5 years of therapy. These findings are consistent with the phase 2b core and extension study findings, wherein a sustained reduction in ARR with ponesimod 20 mg was observed over a long-term treatment of approximately 8 years [9]. Furthermore, both regimens demonstrated long-term efficacy in controlling disease activity, with consistent and sustained reductions in ARR, CUALs, and MRI estimates over an extended duration. Both core study treatment groups demonstrated a low ARR throughout the extension period. While ARR remained low in both groups during the LTE, numerically lower disease activity measures were observed in the continuous ponesimod 20 mg group.

During the extension study, ARR and CUALs trended lower over time, and there were no notable differences in efficacy between the 2 treatment groups. Over the study period, the proportion of participants experiencing 12-week and 24-week CDA was numerically lower in the P20 mg/P20 mg group than in the T14 mg/P20 mg group, suggesting reduced risk of long-term disability progression with continuous ponesimod treatment. These results align with findings from long-term follow-up studies of other DMTs wherein the EDSS scores remained stable over a 10-year period in participants receiving natalizumab or fingolimod [17, 18].

Overall, changes from core baseline in mean absolute EDSS score increased slightly over time, and increases were numerically higher in the T14 mg/P20 mg group compared with the P20 mg/P20 mg group throughout the extension study. Brain volume loss was comparable between groups and consistent with findings from the core OPTIMUM study. MRI data from both groups showed notable reductions in new or enlarging T2 lesions, with a numerically higher proportion of participants remaining free of new lesions for the continuous ponesimod group.

Absence of disease activity, as defined by MRI and clinical measures (NEDA), is a key marker assessing treatment efficacy [19]. CUALs are sensitive MRI biomarkers that detect new or enlarging lesions, offering valuable insight into treatment effectiveness in MS [20]. In this LTE, participants in both groups showed a reduction in cumulative CUALs over time, with a numerically higher proportion of participants in the continuous ponesimod group remaining free of CUALs. This finding is consistent with the results of the long-term phase 2b and core OPTIMUM studies [8, 9]. Additionally, only a self-selected subset of participants who completed the core study entered the LTE, resulting in a limited number of participants with complete data for all NEDA-4 components; therefore, the low proportion of patients achieving NEDA-4 should be interpreted with caution.

There are a few limitations in this study; the open-label design of the extension study could introduce potential biases, as both the participants and investigators were aware of the treatment assignments. Furthermore, the study was not designed to evaluate the effects of treatment interruption or the potential for rebound disease activity following ponesimod discontinuation. The brief protocol-mandated interruption (14 days) between the core study and extension study, coupled with limited follow-up and absence of MRI data consistently collected shortly after the interruption, limits the ability to draw conclusions regarding post-interruption disease activity. Also, the long-term effects of the treatment, including safety outcomes, need to be interpreted with caution due to the lack of a comparator group and the potential for other confounding factors such as selective attrition or treatment switching during the extended follow-up period.

## Conclusion

In this 5-year extension study, ponesimod showed sustained efficacy in reducing ARR and preventing MRI activity in participants with RMS. The study also showed that ponesimod was effective in achieving NEDA-3 and NEDA-4 status, further supporting a sustained control of disease activity. Additionally, the efficacy and safety results for participants after switching from teriflunomide to ponesimod were comparable to those who received ponesimod continuously, suggesting that switching therapies did not compromise the long-term benefits of ponesimod. Consistent with the previous findings, ponesimod demonstrated long-term efficacy with no new safety signals in participants with RMS.

## Supplementary Information

Below is the link to the electronic supplementary material.

Supplementary file1 (PDF 151 KB)

## Data Availability

The data-sharing policy of Johnson & Johnson is available at https://www.innovativemedicine.jnj.com/ourinnovation/clinical-trials/transparency. As noted on this site, requests for access to the study data can be submitted through the Yale Open Data Access [YODA] Project site at https://yoda.yale.edu/.
