# Supplementary material for: Long-term safety and efficacy of ponesimod in participants with relapsing multiple sclerosis: results from the phase 3 OPTIMUM 5-year long term extension study
Source: J Neurol. 2026 Mar 25;273(4):234. doi: 10.1007/s00415-026-13675-7 (PMC13018072; doi:10.1007/s00415-026-13675-7)
Supplement: Supplementary file 1 — Supplementary file1 (PDF 151 KB) [file 415_2026_13675_MOESM1_ESM.pdf]

Supplementary File

Methods

Definition of relapse

A relapse was defined as new, worsening, or recurrent neurological symptoms that occurred at least 30 days after the onset of a preceding relapse, and that lasted at least 24 hours, in the absence of fever or infection. The new, worsening, or recurrent neurological symptoms were to be evaluated by the treating neurologist and, if all the elements of the above definition had been verified, and in the absence of another, better explanation of the participant's symptoms, the event was considered as a relapse. The onset date of the relapse corresponded to the onset date of the symptoms.

A relapse was to be confirmed only when the participant's symptoms were accompanied by an increase in EDSS/FS scores, which was consistent with the participant's symptoms, from a previous clinically stable EDSS/FS assessment (ie, performed at least 30 days after the onset of any previous relapse), obtained by the efficacy assessor and consistent with the following:

- An increase of at least half a step (0.5 points; unless EDSS=0, then an increase of at least 1.0 points was required) or
- An increase of at least 1.0 point in at least 2 FS scores, or
- An increase of at least 2.0 points in at least 1 FS score (excluding bladder/bowel and cerebral).

Results

Supplementary Figure 1: Study design

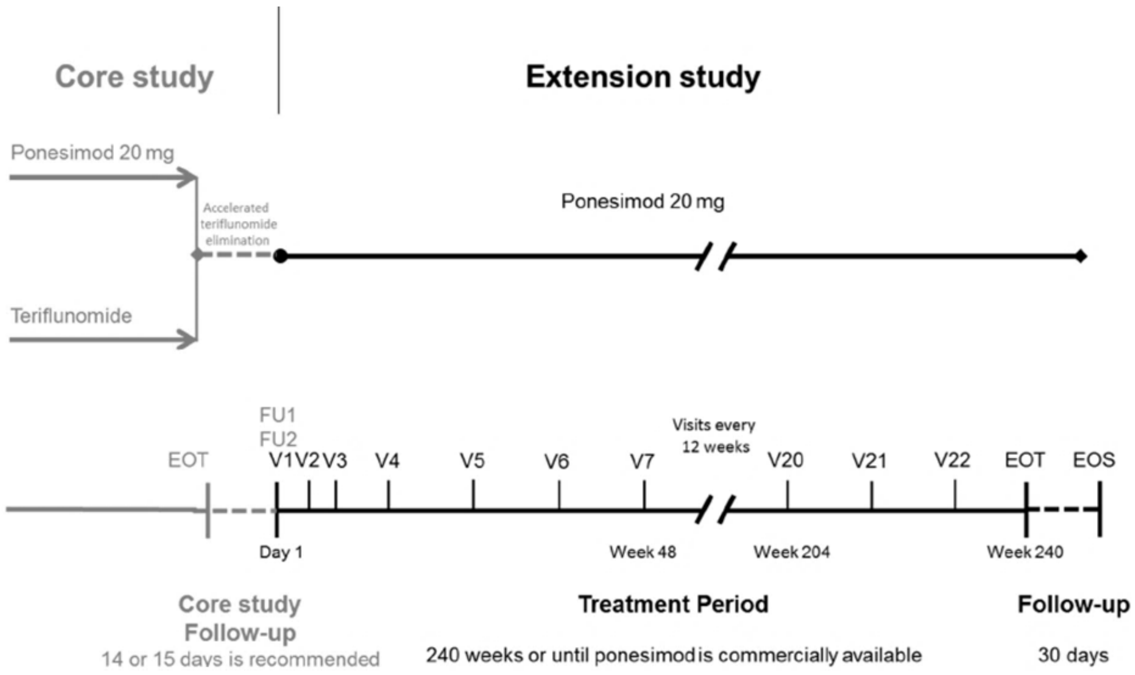

EOS, end-of-study; EOT, end-of-treatment; FU, follow-up; V, visit; - -, no treatment.

Supplementary Figure 2: Participant Disposition

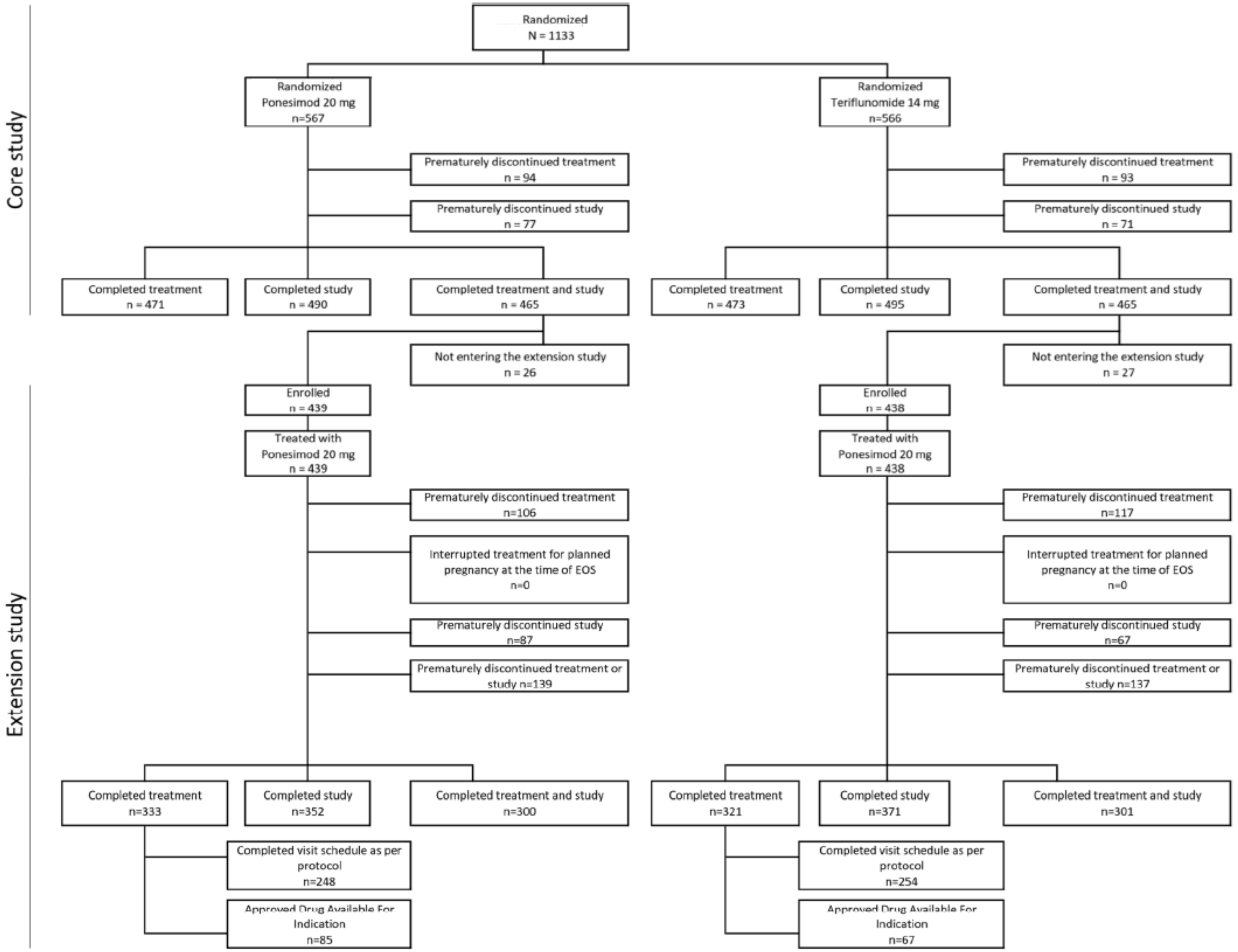

Some data from core study were previously published by Kappos L, et al. *JAMA Neurol*. 2021;78(5):558-567.
